# Quaternary and quinary molecular solids based on structural inequivalence and combinatorial approaches: 2-nitro­resorcinol and 4,6-di­chloro­resorcinol

**DOI:** 10.1107/S2052252520016589

**Published:** 2021-01-11

**Authors:** Madhu Rajkumar, Gautam R. Desiraju

**Affiliations:** a Solid State and Structural Chemistry Unit, Indian Institute of Science, Bangalore 560 012, India

**Keywords:** cocrystal, crystal engineering, supramolecular synthon, intermolecular interaction, multicomponent solid

## Abstract

A synthetic strategy for the formation of stoichiometric quaternary and non­stoichiometric quinary solids is outlined. A series of 2-nitro­resorcinol-based quaternary cocrystals were developed from binary precursors in two conceptual stages. In the first stage, ternary solids are synthesized based on the structural inequivalence at two recognition sites in the binary. In the second stage, the ternary is homologated into a stoichiometric quaternary based on the same concept. Any cocrystal without an inequivalence becomes a synthetic dead end. The combinatorial approach involves lower cocrystal systems with different structural environments and preferred synthon selection from a synthon library in solution. Such are the stepping stones for the isolation of higher cocrystals. In addition, a quaternary cocrystal of 4,6-di­chloro­resorcinol is described wherein an unusual synthon is observed with two resorcinol molecules in a closed loop with two different ditopic bases. The concept of the virtual synthon in binaries with respect to isolated ternaries is validated for the 4,6-di­chloro­resorcinol system. It is possible that only some binary systems are amenable to homologation into higher cocrystals. The reasons for this could have to do with the existence of preferred synthon modules, in other words, the critical components of the putative higher assembly that cannot be altered. Addition of the third and fourth component might be more flexible, and the choices of these com­ponents, possible from a larger pool of chemically related molecules.

## Abstract 2

Stoichiometric ternary and quaternary, and nonstoichiometric quinary molecular solids were engineered based on structural inequivalence and combinatorial approaches from 2-nitroresorcinol. A total of 32 binary, ternary, quaternary and quinary solids were characterized. The great difficulty in obtaining such solids is that crystallization must occur in a single step from precursors in solution, with multistep sequences, as are typical in solution syntheses, not being possible in most situations.

## 1. Introduction   

The design of higher-order multicomponent cocrystals (Bond, 2007 ▸; Bolla & Nangia, 2015 ▸), especially ternaries and quater­naries, is a challenging synthetic exercise (Mir *et al.*, 2019 ▸) requiring a proper knowledge of the robustness of specific supramolecular synthons (Desiraju, 1995 ▸; Shattock *et al.*, 2008 ▸). Any synthetic strategy towards higher cocrystals should be of high supramolecular yield and without contamination from by-products such as binaries and pure compounds. A very limited number of stoichiometric quaternaries (less than 20) have been obtained to date (Bhogala & Nangia, 2008 ▸; Dandela *et al.*, 2018 ▸; Dubey *et al.*, 2016 ▸; Mir *et al.*, 2016 ▸; Paul *et al.*, 2018 ▸; Paul & Desiraju, 2019 ▸). All quaternary cocrystals thus far obtained by our group are based on either structural inequivalences or a combinatorial approach. These higher cocrystal experiments suggest that combinations of resorcinols and methyl­ated pyrazines could be the way forward for general synthetic strategies (Dubey *et al.*, 2016 ▸; Mir *et al.*, 2016 ▸; Paul *et al.*, 2018 ▸; Paul & Desiraju, 2019 ▸). The inclusion of a fifth component stoichiometrically within the same crystal still remains an open challenge because with an increasing number of components and functional groups, the number of possible permutations creates very difficult synthetic challenges (Gunawardana & Aakeröy, 2018 ▸). In this article, we report seven quaternary and two quinary molecular solids to further strengthen the generality of our synthetic strategy towards higher cocrystals.

The structural inequivalence strategy (Mir *et al.*, 2016 ▸) is an outcome of molecule selection by the chemist in that if any particular molecule is bound at two distinct crystallographic environments in a (lower) cocrystal structure, this distinction can be exploited to homologate into a higher cocrystal by substituting a new, but similar, molecule at the more loosely bound site. This structural inequivalence is always integrated with a graded interaction hierarchy (Aakeröy *et al.*, 2001 ▸). On the other hand, the combinatorial strategy (Dubey & Desiraju, 2014 ▸) depends on the fact that if a molecule persists in many forms (polymorphs and/or pseudopolymorphs, the latter being essentially stoichiometric variants), crystallization of such a molecule with another component can lead to a higher cocrystal which is anticipated with a selection of the fittest interactions that are possible from a pool of multiple competing interactions for this particular molecule.

## 2. Results and discussion   

**Figure 1:**
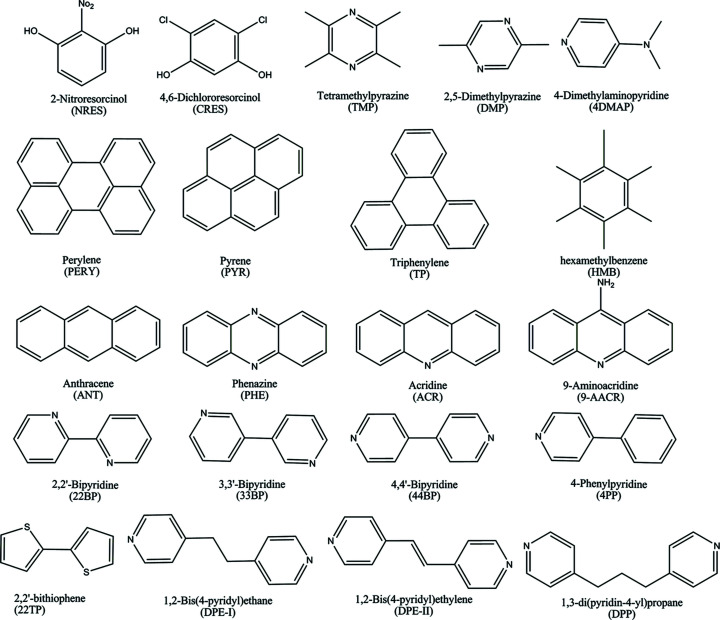
Compounds used in this study with acronyms.

**Figure 2:**
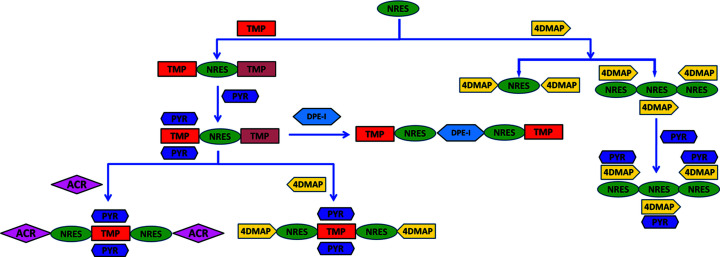
Synthetic strategy for the preparation of the NRES:TMP:PYR:ACR and NRES:TMP:PYR:4DMAP quaternary cocrystals. Colour coding and shapes represent distinct chemical and geometrical features of the molecules.

Our approach to the design of quaternary and quinary molecular solids of 2-nitro­resorcinol (NRES) is based on the simultaneous use of chemical (interaction-based) and geometrical (shape- and size-based) recognition information (Aakeröy *et al.*, 2007 ▸; Moorthy *et al.*, 2010 ▸). The compounds used in this study with acronyms are given in Fig. 1 ▸ and the synthetic strategy involved in the quaternary cocrystal synthesis for NRES:TMP:PYR:ACR and NRES:TMP:PYR:4DMAP is presented in Fig. 2 ▸. In these quaternaries, the binary cocrystal of NRES with tetra­methyl­pyrazine (TMP) is the basic template. In our hands, this binary cocrystal was obtained in two pseudopolymorphic variants. The first is a 2:1 form that crystallizes in the space group *P*1. The second is a 2:3 form which crystallizes in the space group *P* 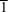. Details of these two cocrystals are given in the supporting information. Because the TMP molecules are located on pseudocentres of symmetry in the noncentrosymmetric 2:1 form, adequate care was taken in the space-group assignment.

**Figure 3:**
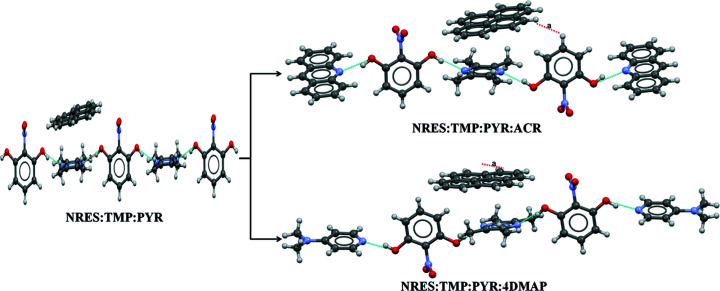
The development of the NRES:TMP:PYR:ACR and NRES:TMP:PYR:4DMAP quaternaries from the NRES:TMP:PYR ternary in which one of the TMP molecules is replaced by a fourth component, ACR or 4DMAP, using O—H⋯N hydrogen bonding.

In any event, the connectivity of the TMP and NRES molecules is the same in both of these cocrystals and, most significantly, the TMP molecule is found in two distinct crystallographic environments in both cases. Of the two TMP molecules, one is connected to NRES only *via* O—H⋯N hydrogen bonds to give an infinite one-dimensional (1D) chain, while the other is additionally involved in weak C—H⋯O, C—H⋯π and π–π interactions with adjacent NRES molecules. As C—H⋯O, C—H⋯π and π–π interactions are comparable in strength, a PYR molecule, which has the capability of forming a C—H⋯π interaction with TMP, may be incorporated as the third component (replacing the NRES that bonds weakly *via* C—H⋯O). In this ternary, and as prescribed, the PYR molecule is connected with one of the two TMP molecules through C—H⋯π interactions, while the other is still unaffected; accordingly, a fourth component, which may be either ACR or 4DMAP, may be introduced with interaction substitution to homologate to the quaternaries NRES:TMP:PYR:ACR and NRES:TMP:PYR:4DMAP (Fig. 3 ▸). In both cases, the monotopic bases ACR and 4DAMAP are connected at the two ends of the assembly but these become synthetic dead ends for homologation into quinaries. In other words, since inequivalences were not found in any of the quaternaries we isolated, stoichiometric inclusion of a fifth component with this strategy is not possible.

While developing these quaternaries, two ternaries were simultaneously obtained, namely, NRES:4DMAP:PYR and NRES:TMP:DPE-I. Let us first consider the former. Crystallization of NRES with 4DMAP gave a 1:1 cocrystal and a 1:1 binary salt (from MeOH and MeCN, respectively). In the cocrystal, the two components are connected *via* O—H⋯.N hydrogen bonding at each hydroxy-group end of NRES; in the salt, the NRES molecules are linked to one another *via* O—H⋯O hydrogen bonds to form a chain, while 4DMAP molecules are connected to them *via* N—H⋯O hydrogen bonds. In the next step, PYR is added to the binary mixture to obtain a ternary in which PYR is stacked with 4DMAP through a C—H⋯π interaction. The other ternary, NRES:TMP:DPE-I, was obtained when the ternary mixture NRES:TMP:PYR was crystallized with DPE-I (to obtain a quaternary). This procedure removed the PYR molecule and an entirely different supramolecular topology was obtained in the resulting ternary. In this cocrystal, DPE-I is O—H⋯N hydrogen bonded at its two ends to NRES, which uses its other hy­droxy group to connect with TMPs, which terminates the sequence (the second heterocyclic N atom of TMP is ‘free’). Employing the structural inequivalence approach, Komisarek *et al.* (2020 ▸) have reported this kind of synthon in the system 2-methyl­resorcinol--TMP--DPE-I. In both of these ternaries (NRES:4DMAP:PYR and NRES:TMP:DPE-I), no inequivalences are observed at any of the chemically identical sites and therefore these cannot be exploited for further homologation. Both ternaries are synthetic dead ends.

**Figure 4:**
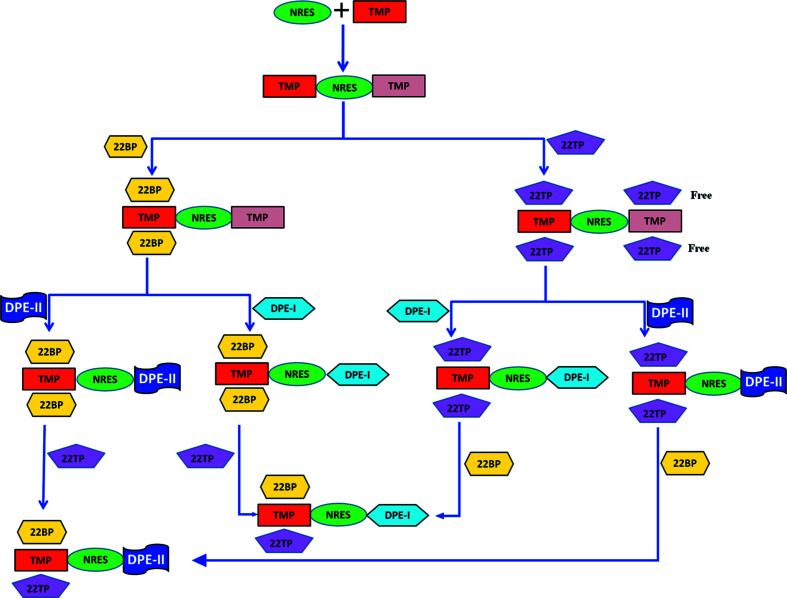
Proposed schemes for the construction of five-component systems.

Let us next consider the synthetic strategy towards quinary cocrystals from quaternaries (Fig. 4 ▸). Here, the binary NRES:TMP (mentioned previously) is crystallized with either 2,2′-bi­pyridine (22BP) or 2,2′-bi­thio­phene (22TBP) to give the ternaries NRES:TMP:22BP and NRES:TMP:22TBP in 1:2:2 and 1:2:4 stoichiometries, respectively. In these ternary systems, it is found that the two TMP molecules flanking NRES are in different crystallographic environments (as seen previously with PYR). In NRES:TMP:22BP, of the two TMP molecules, one forms C—H⋯π interactions with 22BP, while the other is not involved in any such interaction; accordingly, the latter may be replaced by other ditopic N-atom donors. In NRES:TMP:22TBP, there is a similar situation. These differences are sufficient for the replacement of such ‘free’ molecules by another N-atom donor. Accordingly, in the next homologation step, each of these ternaries was crystallized with DPE-I or DPE-II, so that we successfully obtained four 1:1:1:1 quaternaries, namely, NRES:TMP:22BP:DPE-I and NRES:TMP:22BP:DPE-II with 22BP, and cor­respondingly NRES:TMP:22TBP:DPE-I and NRES:TMP:22TBP:DPE-II with 22TBP. These quaternaries are, however, syn­thetic dead ends and structural inequivalences may no longer be used to homologate to the quinary stage.

**Figure 5:**
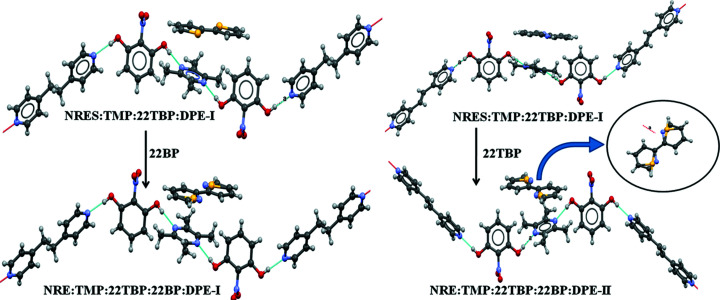
Quinaries NRES:TMP:22TBP:22BP:DPE-I and NRE:TMP:22TBP:22BP:DPE-II. Note that the fourth and fifth components are included in a solid-solution manner.

A careful analysis of these quaternary structures paved the way to include a fifth component in these systems. In this context, the phenyl/thiophenyl exchange rule is relevant (Thallapally *et al.*, 2000 ▸; Tothadi *et al.*, 2011 ▸). According to this concept, 2,2′-bi­thio­phene, 2,2′-bi­phenyl and 2,2′-bi­pyridine are volume equivalents (–S– ≃ –CH=CH– ≃ –CH=N–) and so are mutually exchangeable in a crystal structure. The question arises therefore as to whether it might be possible to obtain quinary cocrystals if *both* 2,2′-bi­thio­phene and 2,2′-bi­pyridine are taken together along with the other three components NRES:TMP:DPE-I. Accordingly, we ground NRES, TMP and DPE-I with 0.5:0.5 ratios of 22BP and 22TBP, and isolated a five-component solid-solution cocrystal (Fig. 5 ▸). We carried out a similar crystallization experiment with DPE-II and obtained the corresponding five-component solid-solution cocrystal NRES:TMP:22BP:22TBP:DPE-II.

**Figure 6:**
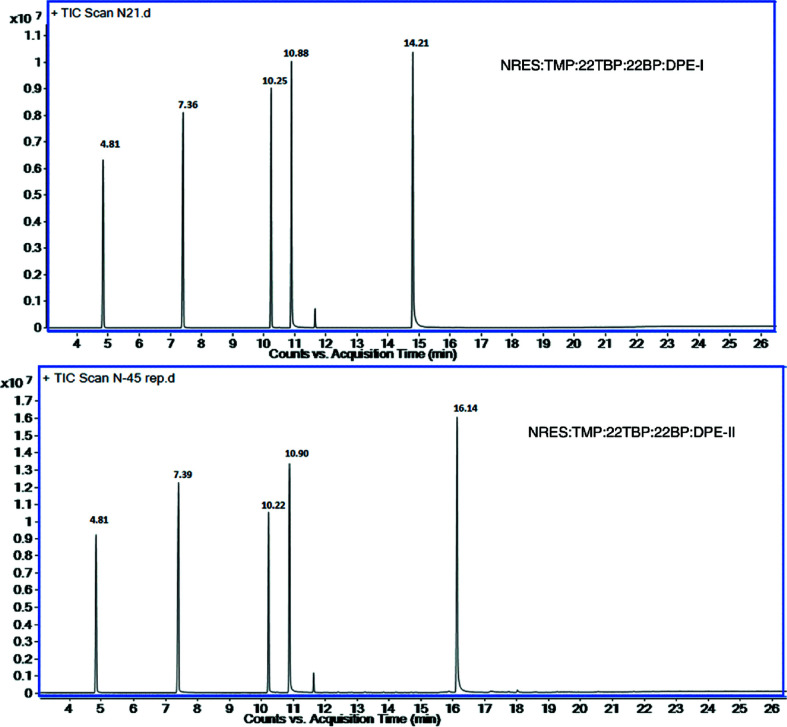
GCMS spectra of five-component systems.

To confirm the existence of five different organic molecules in the same crystal, we carried out GC–MS (gas chromatography–mass spectrometry) analysis. For this, single crystals of the quinary system were dissolved in MeOH. We observed five different peaks at different retention times (Fig. 6 ▸; the very small peak is an unknown impurity, possibly from stopcock grease).

**Figure 7:**
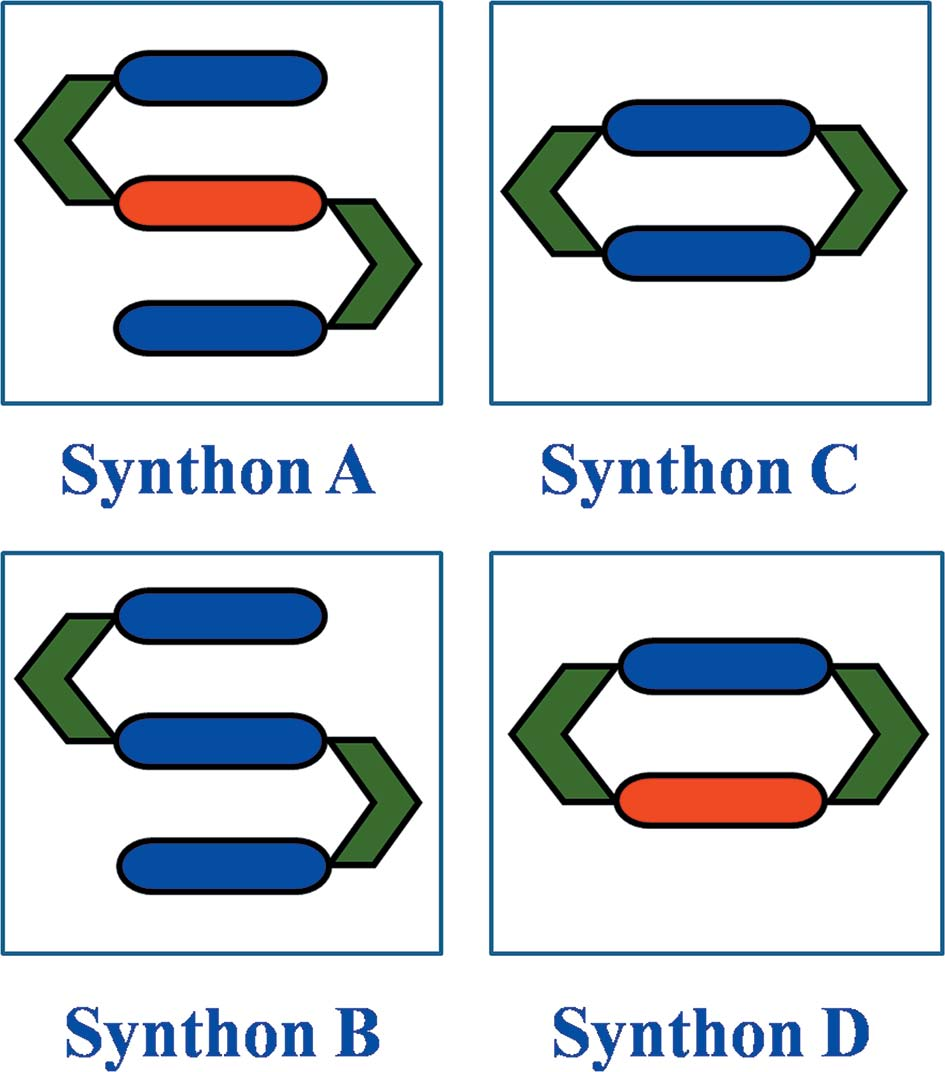
Possible supramolecular synthons of the 4,6-di­chloro­resorcinol system with N-atom donors. CRES is shown in green and blue and red are ditopic bases. Note that synthon A (seen elsewhere) is not observed in this study. Synthon D, which is unsymmetrical, is rarely seen with resorcinols in general (Paul & Desiraju, 2019 ▸).

**Figure 8:**
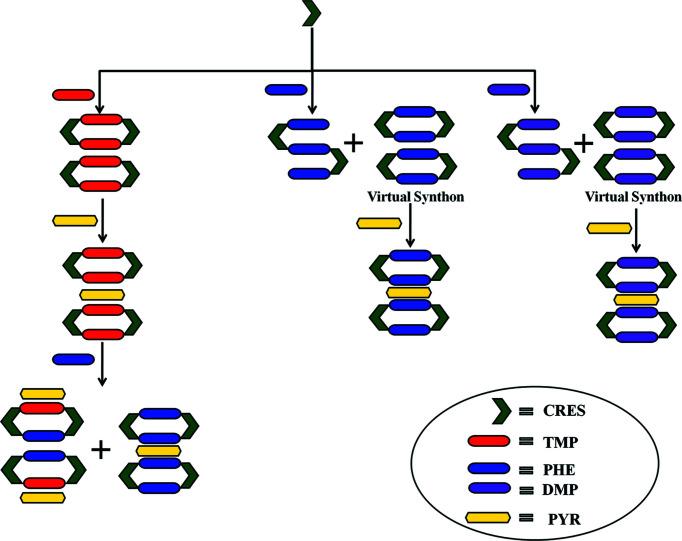
A cartoon representation of the synthetic strategy for the CRES quaternary. Distinct chemical species are given with different shapes and in different colours.

We now discuss the combinatorial synthetic strategy based on long-range synthon Aufbau modules (LSAM) in the higher cocrystals of 4,6-di­chloro­resorcinol. The modular nature of the interactions may be utilized to obtain higher cocrystals by adding and/or exchanging a new molecule in the LSAM of the lower cocrystals. A putative supramolecular synthon of 4,6-di­chloro­resorcinol with complementary N-atom donors is illustrated in Fig. 7 ▸ and a schematic representation of the synthesis of quaternary cocrystals is shown in Fig. 8 ▸.

**Figure 9:**
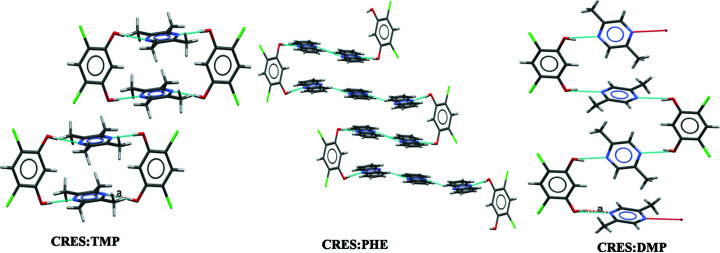
Binary cocrystals with synthons B and C. Note that CRES:PHE shows an unusual open synthon.

In this attempt, we obtained three each of the binary and ternary cocrystals, and one quaternary cocrystal. Crystallization of CRES with (one of) TMP, PHE or DMP resulted in a stoichiometric binary cocrystal. In the 1:1 CRES:TMP cocrystal, the TMP molecules form discrete synthon C (closed CRES:TMP tetramer synthons, the so-called MacGillivray synthon) (Papaefstathiou *et al.*, 2001 ▸; Ghorai *et al.*, 2013 ▸) that are laterally offset with respect to one another. Next, we observed the formation of an unusual open synthon in the 1:3 CRES:PHE binary in which one hydroxy group of the CRES molecule is connected to two PHE molecules through O—H⋯N hydrogen bonding, followed by C—H⋯N interactions, while the second hydroxy group of CRES is linked to three PHE molecules so as to form a continuous 1D LSAM (Fig. 9 ▸). These open synthons are further stacked with π–π interactions between PHE molecules. The CRES:DMP binary forms another kind of continuous 1D LSAM in which the two DMP molecules that are connected to CRES are perpendicular to each other, leading to C—H⋯π interactions. In all these binaries, we found that there are some weak interactions (C—H⋯O, π—π and C—H⋯π) which may be opened further to obtain higher cocrystals.

**Figure 10:**
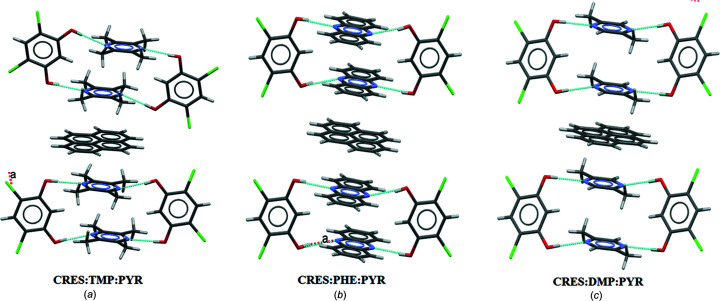
The 2:2:1 stoichiometric ternary cocrystals with synthon C, *i.e.* (*a*) CRES:TMP:PYR, (*b*) CRES:PHE:PYR and (*c*) CRES:DMP:PYR.

**Figure 11:**
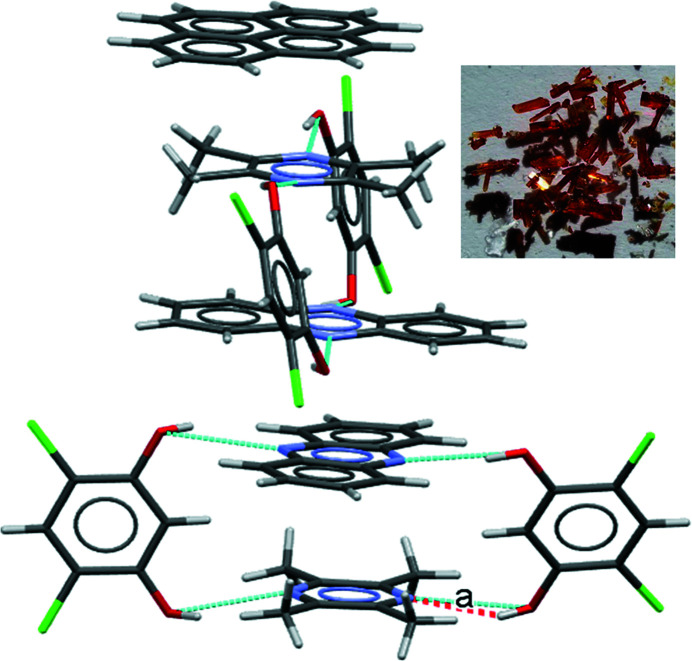
The structure of the quaternary cocrystal CRES:PHE:TMP:PYR with the unusual synthon D. The inset photograph shows the simultaneous growth of the quaternary (red) and ternary (yellow) crystals from a solution containing CRES, PHE, TMP and PYR.

Accordingly, in the next homologation experiment, the PYR molecule was incorporated within these binaries and this resulted in three distinct ternaries. Interestingly, although binaries with closed synthon C were not isolated (say in CRES:PHE and CRES:DMP), all ternaries in this system have the closed synthon C wherein both bases are the same (Fig. 10 ▸). This suggests the idea of a *virtual synthon* which, while it exists in solution, is only manifested in some isolated cocrystals in the entire system. Such a synthon is virtual with respect to binary crystallization and becomes real with respect to ternary systems (Dubey & Desiraju, 2014 ▸). Exploiting the similarities of the synthons in these ternary systems, three attempts at homologation were made: (i) CRES:TMP:PYR with PHE; (ii) CRES:TMP:PYR with DMP; and (iii) CRES:DMP:PYR with PHE. In the process of crystallizing CRES:TMP:PYR with PHE, two distinguishable crystals differing in colour (red and yellow) were obtained from the same solution. The red crystal was identified as the ternary CRES:PHE:PYR and the yellow crystal as the quaternary CRES:TMP:PYR:PHE (Fig. 11 ▸). This quaternary cocrystal contains the anomalous MacGillivray synthon (Paul & Desiraju, 2019 ▸) (synthon D) with two different bases (TMP and PHE). Crystallization according to protocols (ii) and (iii) above did not prove fruitful. The crystallographic details of all the cocrystals used are provided in the supporting information.

## 3. Conclusions   

The results obtained in this work validate the structural inequivalence and combinatorial approaches for the isolation of quaternary molecular solids and the use of such approaches combined with solid-solution methods for the achieving of quinary solids, providing more synthetic generality. In the present study, six stoichiometric quaternaries and two non­stoichiometric quinary cocrystals are reported based on the structural inequivalence approach. The two-component combinations of TMP:PYR, TMP:22BP and TMP:22TBP play a pivotal role in the formation of quaternary cocrystals in which there are C—H⋯π interactions. In the case of the NRES:TMP:PYR ternary, monotopic N-atom donors are required for the formation of quaternary cocrystals, whereas in the case of NRES:TMP:22BP and NRES:TMP:22TBP, symmetrical ditopic N-atom donors are required. After reaching synthetic dead ends at the quaternary level, the concept of shape–size similarity was utilized as a synthetic tool for obtaining five-component systems. In addition, an interesting quaternary cocrystal was obtained based on the combinatorial approach in which an unusual tetramer synthon, containing two different bases, was obtained. Finally, a closed supramolecular synthon, which was not observed in binary cocrystals, appears at the ternary and quaternary levels, strengthening the concept of virtual synthons in solution.

The formation of higher cocrystals (ternary and higher) poses restrictions on exactly what molecular precursors are chosen, and on what exactly are the experimental conditions of the crystallization experiment. Many of these choices may be, in the end, arbitrary, especially regarding the solvents chosen and the temperatures and cooling rates employed. Of course, the latter experimental variables are critical in many crystallizations, including in some of the simplest single-substance experiments, as they depend on the details of the relevant solvent–solute phase diagrams. What is of relevance here is to ascertain if certain compounds or types of compounds are more amenable to the formation of higher cocrystals than others. Expectedly, polyfunctional compounds such as diphenols, triphenols and ditopic bases were found to be suitable. In the course of our work in this area over the past decade, we have found that a combination of a 2-substituted (or other) resorcinol with TMP is generally required to form ternary and higher cocrystals. There are a few exceptions but the above is largely true across many crystallization experiments that have led to the isolation and characterization of around 75 ternaries and 20 quaternaries. The present study is no exception to these general trends. There is some latitude in the nature of the 2-substituent in the resorcinol component (or the other substituents) for higher cocrystals to be formed but *TMP seems to be necessary*. It is seen that both tri­methyl­pyrazine and 2,5-di­methyl­pyrazine form binaries with 2-sub­stituted resorcinols, but these cannot be homologated. On the other hand, there is considerable flexibility in the choice of the third and fourth component once resorcinol and TMP are selected as two of the components in the crystallization experiment. These can be chosen from several heterocyclic and nonheterocyclic bases, and from condensed ring hydro­carbons that are usually C—H⋯π stacked on the TMP.

These results suggest the relevance of the resorcinol–TMP association, which seems to be the basic building block of higher architectures through synthons of types A through D (Fig. 7 ▸). Why such synthons are important for homologation is not clear, and especially why TMP itself seems to be so important is unknown. It is possible that these small synthons can expand easily into LSAMs that incorporate the third and fourth component, but again the specificity of synthons A through D is not easy to rationalize. More high-throughput crystallizations involving combinatorial and robotic methods may prove to be the route to expanding the structural space occupied by stoichiometric ternary and quaternary cocrystals. However, what seems clear enough is that there exist a vast number of higher cocrystals that remain to be discovered.

## Supplementary Material

Crystal structure: contains datablock(s) 1NRES-TMP, 2NRES-TMP, 3NRES-ACR, 4NRES-DMP, 5NRES-9AACR, 6NRES-33BP, 7NRES-44BP, 8NRES-DPE-I, 9NRES-DPE-II, 10NRES-44AZO, 11NRES-4DMAP, 12NRES-4DMAP-II, 13NRES-TMP-PYR, 14NRES-4DMAP-PYR, 15NRES-TMP-DPE-I, 16NRES-TMP-22BP, 17NRE-TMP-22TBP, 18NRES-TMP-PYR-ACR, 19NTES-TMP-PYR-4DMAP, 20NRES-TMP-22BP-DPE-I, 21NRES-TMP-22BP-DPE-II, 22NRES-TMP-22TBP-DPE-I, 23NRES-TMP-22TBP-DPE-II, 24NRES-TMP-22BP-22TBP-DPE-I, 25NRES-TMP-22BP-22TBP-DPE-II, 26CRES-TMP, 27CRES-PHE, 28CRES-DMP, 29CRES-TMP-PYR, 30CRES-PHE-PYR, 31CRES-DMP-PYR, 32CRES-TMP-PHE-PYR. DOI: 10.1107/S2052252520016589/yc5029sup1.cif

Crystallization experimental details, characterization techniques, GC-MS details and spectra, and description of the two pseudopolymorphic binary cocrystals NRES:TMP. DOI: 10.1107/S2052252520016589/yc5029sup2.pdf

Structure factors: contains datablock(s) 1NRES-TMP. DOI: 10.1107/S2052252520016589/yc50291NRES-TMPsup3.hkl

Structure factors: contains datablock(s) 2NRES-TMP. DOI: 10.1107/S2052252520016589/yc50292NRES-TMPsup4.hkl

Structure factors: contains datablock(s) 3NRES-ACR. DOI: 10.1107/S2052252520016589/yc50293NRES-ACRsup5.hkl

Structure factors: contains datablock(s) 4NRES-DMP. DOI: 10.1107/S2052252520016589/yc50294NRES-DMPsup6.hkl

Structure factors: contains datablock(s) 5NRES-9AACR. DOI: 10.1107/S2052252520016589/yc50295NRES-9AACRsup7.hkl

Structure factors: contains datablock(s) 6NRES-33BP. DOI: 10.1107/S2052252520016589/yc50296NRES-33BPsup8.hkl

Structure factors: contains datablock(s) 7NRES-44BP. DOI: 10.1107/S2052252520016589/yc50297NRES-44BPsup9.hkl

Structure factors: contains datablock(s) 8NRES-DPE-I. DOI: 10.1107/S2052252520016589/yc50298NRES-DPE-Isup10.hkl

Structure factors: contains datablock(s) 9NRES-DPE-II. DOI: 10.1107/S2052252520016589/yc50299NRES-DPE-IIsup11.hkl

Structure factors: contains datablock(s) 10NRES-44AZO. DOI: 10.1107/S2052252520016589/yc502910NRES-44AZOsup12.hkl

Structure factors: contains datablock(s) 11NRES-4DMAP. DOI: 10.1107/S2052252520016589/yc502911NRES-4DMAPsup13.hkl

Structure factors: contains datablock(s) 12NRES-4DMAP-II. DOI: 10.1107/S2052252520016589/yc502912NRES-4DMAP-IIsup14.hkl

Structure factors: contains datablock(s) 13NRES-TMP-PYR. DOI: 10.1107/S2052252520016589/yc502913NRES-TMP-PYRsup15.hkl

Structure factors: contains datablock(s) 14NRES-4DMAP-PYR. DOI: 10.1107/S2052252520016589/yc502914NRES-4DMAP-PYRsup16.hkl

Structure factors: contains datablock(s) 15NRES-TMP-DPE-I. DOI: 10.1107/S2052252520016589/yc502915NRES-TMP-DPE-Isup17.hkl

Structure factors: contains datablock(s) 16NRES-TMP-22BP. DOI: 10.1107/S2052252520016589/yc502916NRES-TMP-22BPsup18.hkl

Structure factors: contains datablock(s) 17NRE-TMP-22TBP. DOI: 10.1107/S2052252520016589/yc502917NRE-TMP-22TBPsup19.hkl

Structure factors: contains datablock(s) 18NRES-TMP-PYR-ACR. DOI: 10.1107/S2052252520016589/yc502918NRES-TMP-PYR-ACRsup20.hkl

Structure factors: contains datablock(s) 19NTES-TMP-PYR-4DMAP. DOI: 10.1107/S2052252520016589/yc502919NTES-TMP-PYR-4DMAPsup21.hkl

Structure factors: contains datablock(s) 20NRES-TMP-22BP-DPE-I. DOI: 10.1107/S2052252520016589/yc502920NRES-TMP-22BP-DPE-Isup22.hkl

Structure factors: contains datablock(s) 21NRES-TMP-22BP-DPE-II. DOI: 10.1107/S2052252520016589/yc502921NRES-TMP-22BP-DPE-IIsup23.hkl

Structure factors: contains datablock(s) 22NRES-TMP-22TBP-DPE-I. DOI: 10.1107/S2052252520016589/yc502922NRES-TMP-22TBP-DPE-Isup24.hkl

Structure factors: contains datablock(s) 23NRES-TMP-22TBP-DPE-II. DOI: 10.1107/S2052252520016589/yc502923NRES-TMP-22TBP-DPE-IIsup25.hkl

Structure factors: contains datablock(s) 24NRES-TMP-22BP-22TBP-DPE-I. DOI: 10.1107/S2052252520016589/yc502924NRES-TMP-22BP-22TBP-DPE-Isup26.hkl

Structure factors: contains datablock(s) 25NRES-TMP-22BP-22TBP-DPE-II. DOI: 10.1107/S2052252520016589/yc502925NRES-TMP-22BP-22TBP-DPE-IIsup27.hkl

Structure factors: contains datablock(s) 26CRES-TMP. DOI: 10.1107/S2052252520016589/yc502926CRES-TMPsup28.hkl

Structure factors: contains datablock(s) 27CRES-PHE. DOI: 10.1107/S2052252520016589/yc502927CRES-PHEsup29.hkl

Structure factors: contains datablock(s) 28CRES-DMP. DOI: 10.1107/S2052252520016589/yc502928CRES-DMPsup30.hkl

Structure factors: contains datablock(s) 29CRES-TMP-PYR. DOI: 10.1107/S2052252520016589/yc502929CRES-TMP-PYRsup31.hkl

Structure factors: contains datablock(s) 30CRES-PHE-PYR. DOI: 10.1107/S2052252520016589/yc502930CRES-PHE-PYRsup32.hkl

Structure factors: contains datablock(s) 31CRES-DMP-PYR. DOI: 10.1107/S2052252520016589/yc502931CRES-DMP-PYRsup33.hkl

Structure factors: contains datablock(s) 32CRES-TMP-PHE-PYR. DOI: 10.1107/S2052252520016589/yc502932CRES-TMP-PHE-PYRsup34.hkl

CCDC references: 1989214, 1989215, 1989216, 1989217, 1989218, 1989219, 1989220, 1989221, 1989222, 1989223, 1989224, 2026215, 2026216, 2026217, 2026218, 2026219, 2026220, 2026221, 2026222, 2026223, 2026224, 2026225, 2026226, 2026227, 2026228, 2026229, 2026230, 2026231, 2026232, 2026233, 2026234, 2039064
