# Supplementary material for: Quaternary and quinary molecular solids based on structural inequivalence and combinatorial approaches: 2-nitro­resorcinol and 4,6-di­chloro­resorcinol
Source: IUCrJ. 2021 Jan 11;8(Pt 2):178–85. doi: 10.1107/S2052252520016589 (PMC7924232; doi:10.1107/S2052252520016589)
Supplement: Supplementary file 2 [file m-08-00178-sup2.pdf]

IUCrJ

Volume 8 (2021)

Supporting information for article:

Quaternary and quinary molecular solids based on structural inequivalence and combinatorial approaches: 2-nitroresorcinol and 4,6-dichlororesorcinol

Rajkumar Madhu and Gautam R, Desiraju

Quaternary and quinary molecular solids based on structural inequivalence and combinatorial approaches: 2-Nitroresorcinol and 4,6-Dichlororesorcinol

Rajkumar Madhu[a] and Gautam R, Desiraju[a]*

[a]Solid State and Structural Chemistry Unit, Indian Institute of Science, Bangalore, India, 560 012

Correspondence email: gdesiraju@iisc.ac.in

S1. Crystallization experimental details

The solvent assisted grinding procedure was adopted for the crystallization of the multi-component molecular cocrystals. Using a mortar and a pestle, solid compounds (in definite stoichiometric ratios) were taken together with a few drops of solvent and ground. The same process was repeated several times to get a homogeneous mixture and then the mixture was dissolved in different solvents and kept aside undisturbed for crystallization. Solvents used are methanol (MeOH), acetonitrile (MeCN), tetrahydrofuran (THF), nitromethane ($MeNO_2$), acetone (Me_2CO), ethylacetate (EtOAc) and benzene, and mixtures there of as specified in individual cases. It is to be noted that when the stoichiometric quantities of materials were taken and ground together in these crystallization experiments, the crystals obtained were largely or exclusively of the one desired cocrystal phase.

2-Nitroresorcinol.Tetramethylpyrazine (Form-I) (1): A 2:1 molar ratio of 2-nitroresorcinol and tetramethylpyrazine along with 2-3 drops of MeOH was ground in a mortar with a pestle for 15 minutes. After 2-3 days, good diffraction quality pale yellow colour crystals were harvested from MeOH at room temperature.

2-Nitroresorcinol.Tetramethylpyrazine (Form-II) (2): A 2:3 molar ratio of 2-nitroresorcinol and tetramethylpyrazine along with 2-3 drops of MeOH was ground in a mortar with a pestle for 15 minutes. After 2-3 days, good diffraction quality pale yellow colour crystals were obtained from THF at room temperature.

2-Nitroresorcinol.2,5-Dimethylpyrazine (3): An equimolar ratio of 2-nitroresorcinol and 2,5-dimethylpyrazine along with 2-3 drops of MeOH was ground in a mortar with a pestle until evaporation of the solvent. Yellow colour block shaped diffraction quality crystals were obtained after 3-4 days from MeCN.

2-Nitroresorcinol.Acridine (4): A 1:1 mixture of 2-nitroresorcinol and acridine along with 2-3 drops of MeOH was ground in a mortar with a pestle for 15 minutes. Yellow colour block shaped diffraction quality crystals were obtained after 3-5 days from THF.

2-Nitroresorcinol.9-Aminoacridine (5): A 1:1 mixture of 2-nitroresorcinol and 9-aminoacridine along with 2-3 drops of MeOH was ground in a mortar with a pestle for 15 minutes. Yellow coloured block shaped diffraction quality crystals were obtained after 5 days from 1:1 EtOH-Me_2CO.

2-Nitroresorcinol.3,3'-Bipyridine (6): A 1:1 molar ratio of 2-nitroresorcinol and 3,3'-bipyridine along with 2-3 drops of MeOH was ground in a mortar with a pestle for 15 minutes. Diffraction quality block shaped colourless crystals were obtained after 3-4 days from MeCN at room temperature.

2-Nitroresorcinol.4,4'-Bipyridine (7): An equimolar ratio of 2-nitroresorcinol and 4,4'-bipyridine along with 2-3 drops of MeOH was ground in a mortar with a pestle for 15 minutes. Diffraction quality block shaped colourless crystals were obtained after 3-4 days from 1:1EtOAc:THF at room temperature.

2-Nitroresorcinol.1,2-Bis(4-pyridyl)ethane (8): An equimolar ratio of 2-nitroresorcinol and 1,2-bis(4-pyridyl)ethane along with 2-3 drops of MeOH was ground in a mortar with a pestle for 15 minutes. After 3-4 days, light yellow colour block shaped diffraction quality crystals were obtained from MeOH.

2-Nitroresorcinol.1,2-Bis(4-pyridyl)ethene (9): An equimolar ratio of 2-nitroresorcinol and 1,2-bis(4-pyridyl)ethene along with 2-3 drops of MeOH was ground in a mortar with a pestle for 15 minutes. After 3-4 days, light yellow colour block shaped diffraction quality crystals were obtained from MeOH.

2-Nitroresorcinol.4,4′-Azopyridine (10): An equimolar ratio of 2-nitroresorcinol and 4,4′-azopyridine along with 2-3 drops of MeOH was ground in a mortar with a pestle for 15 minutes. Brown colour block shaped diffraction quality crystals were obtained after 3-4 days from MeOH.

2-Nitroresorcinol.4-Dimethylaminopyridine (Form-I) (11): A 1:2 mixture of 2-nitroresorcinol and 4-dimethylaminopyridine along with 2-3 drops of MeOH was ground in a mortar with a pestle for 15 minutes. Brown colour block shaped diffraction quality crystals were obtained after 3-5 days from MeOH.

2-Nitroresorcinol.4-Dimethylaminopyridine (Form-II) (12): An equimolar ratio of 2-nitroresorcinol and 4-dimethylaminopyridine along with 2-3 drops of MeOH was ground in a mortar with a pestle for 15 minutes. Brown colour block shaped diffraction quality crystals were obtained after few days from $MeNO_2$.

2-Nitroresorcinol.Tetramethylpyrazine.Pyrene (13): An equimolar ratio of 2-nitroresorcinol, tetramethylpyrazine and pyrene along with 2-3 drops of MeOH was ground in a mortar with a pestle for 15 minutes. Light yellow colour block shaped diffraction quality crystals were obtained after 3-4 days from MeOH.

2-Nitroresorcinol.4-Dimethylaminopyridine.Pyrene (14): An equimolar ratio of 2-nitroresorcinol, 4-dimethylaminopyridine and pyrene along with 2-3 drops of MeOH was ground in a mortar with a pestle for 15 minutes. Light yellow colour block shaped diffraction quality crystals were obtained after 3-4 days from MeOH.

2-Nitroresorcinol.Tetramethylpyrazine.1,2-Bis(4-pyridyl)ethane (15): A 2:2:1 mixture of 2-nitroresorcinol, tetramethylpyrazine and 1,2-bis(4-pyridyl)ethane along with 2-3 drops of MeOH was ground in a mortar with a pestle for 15 minutes. Pale yellowcolour block shaped diffraction quality crystals were obtained after 4-5 days from MeCN.

2-Nitroresorcinol.Tetramethylpyrazine.2,2'-Bipyridine (16): A 2:2:1 mixture of 2-nitroresorcinol, tetramethylpyrazine and 2,2'-bipyridine along with 2-3 drops of MeOH was ground in a mortar with a pestle for 15 minutes. Yellow colour block shaped diffraction quality crystals were obtained after 3-5 days from MeCN.

2-Nitroresorcinol.Tetramethylpyrazine.2,2'-Bithiophene (17): An equimolar mixture of 2-nitroresorcinol, tetramethylpyrazine and 2,2'-bithiophene along with 2-3 drops of MeOH was ground in a mortar with a pestle for 15 minutes. Yellow colour block shaped diffraction quality crystals were obtained after few days from MeCN.

2-Nitroresorcinol.Tetramethylpyrazine.Pyrene.Acridine (18): A 3:2:2:2 mixture of 2-nitroresorcinol, tetramethylpyrazine, pyrene and acridine along with 2-3 drops of MeOH was ground in a mortar with a pestle for 15 minutes. Pale yellow colour block shaped diffraction quality crystals were obtained after 3-5 days from $MeNO_2$.

2-Nitroresorcinol.Tetramethylpyrazine.Pyrene.4-Dimethylaminopyridine (19): A 2:2:2:1 mixture of 2-nitroresorcinol, tetramethylpyrazine, pyrene and 4-dimethylaminopyridine along with 2-3 drops of MeOH was ground in a mortar with a pestle for 15 minutes. Yellow colour block shaped diffraction quality crystals were obtained after 3-4 days from MeCN.

2-Nitroresorcinol.Tetramethylpyrazine.2,2'-Bipyridine.1,2-Bis(4-pyridyl)ethane (20): A 2:1:1:1 mixture of 2-nitroresorcinol, tetramethylpyrazine, 2,2'-bipyridine and 1,2-bis(4-pyridyl)ethane along with 2-3 drops of MeOH was ground in a mortar with a pestle for 15 minutes. After 3-4 days, block shaped diffraction quality colourless crystals were obtained from $MeNO_2$.

2-Nitroresorcinol.Tetramethylpyrazine.2,2'-Bipyridine.1,2-Bis(4-pyridyl)ethene (21): A 2:1:1:1 mixture of 2-nitroresorcinol, tetramethylpyrazine, 2,2'-bipyridine and 1,2-bis(4-pyridyl)ethene along

with 2-3 drops of MeOH was ground in a mortar with a pestle for 15 minutes. After 3-4 days,block shaped diffraction quality colourless crystals were obtained from $MeNO_2$.

2-Nitroresorcinol.Tetramethylpyrazine.2,2'-Bithiophene.1,2-Bis(4-pyridyl)ethane (22): A 3:2:2:2 mixture of 2-nitroresorcinol, tetramethylpyrazine, 2,2'-bithiophene and 1,2-bis(4-pyridyl)ethane along with 2-3 drops of MeOH was ground in a mortar with a pestle for 15 minutes. Colourless block shaped diffraction quality crystals were obtained after 3-5 days from $MeNO_2$.

2-Nitroresorcinol.Tetramethylpyrazine.2,2'-Bithiophene.1,2-Bis(4-pyridyl)ethene (23): A 3:2:2:2 mixture of 2-nitroresorcinol, tetramethylpyrazine, 2,2'-bisthiophene and 1,2-bis(4-pyridyl)ethene along with 2-3 drops of MeOH was ground in a mortar with a pestle for 15 minutes. Colourless block shaped diffraction quality crystals were obtained after 3-5 days from $MeNO_2$.

2-Nitroresorcinol.Tetramethylpyrazine.2,2'-Bipyridine.2,2'-Bithiophene.1,2-Bis(4-pyridyl)ethane (24): A 2:1:1:1:1 mixture of 2-nitroresorcinol, tetramethylpyrazine, 2,2'-bipyridine, 2,2'-bithiophene and 1,2-bis(4-pyridyl)ethane along with 2-3 drops of MeOH was ground in a mortar with a pestle for 15 minutes. Diffraction quality block shaped colourless crystals were obtained after 3-4 days from THF at room temperature.

2-Nitroresorcinol.Tetramethylpyrazine.2,2'-Bipyridine.2,2'-Bithiophene.1,2-Bis(4-pyridyl)ethene (25): A 2:1:1:1:1 mixture of 2-nitroresorcinol, tetramethylpyrazine, 2,2'-bipyridine, 2,2'-bithiophene and 1,2-bis(4-pyridyl)ethene along with 2-3 drops of MeOH was ground in a mortar with a pestle for 15 minutes. Diffraction quality block shaped colourless crystals were obtained from THF at room temperature after 3-4 days.

4,6-Dichlororesorcinol.Tetramethylpyrazine (26): An equimolar ratio of 4,6-dichlororesorcinol and tetramethylpyrazine along with 2-3 drops of MeOH was ground in a mortar with a pestle for 15 minutes. Diffraction quality block shaped colourless crystals were obtained from MeOH at room temperature after 3-4 days.

4,6-Dichlororesorcinol.Phenazine (27): A 1:1 mixture of 4,6-dichlororesorcinol and phenazine along with 2-3 drops of MeOH was ground in a mortar with a pestle for 15 minutes. Diffraction quality block shaped colourless crystals were obtained from 1:1 EtOH:Me_2CO at room temperature after 3-4 days.

4,6-Dichlororesorcinol.2,5-Dimethylpyrazine (28): A 1:1 molar ratio of 4,6-dichlororesorcinol and 2,5-dimethylpyrazine along with 2-3 drops of MeOH was ground in a mortar with a pestle for 15 minutes. Diffraction quality block shaped colourless crystals were obtained from $MeNO_2$ at room temperature after 3-4 days.

4,6-Dichlororesorcinol.Tetramethylpyrazine.Pyrene (29): A 2:2:1 mixture of 4,6-dichlororesorcinol, tetramethylpyrazine and pyrene along with 2-3 drops of MeOH was ground in a

mortar with a pestle for 15 minutes. Diffraction quality block shaped colourless crystals were obtained from $MeNO_2$ at room temperature after 3-4 days.

4,6-Dichlororesorcinol.Phenazine.Pyrene (30): A 1:1:2 mixture of 4,6-dichlororesorcinol, phenazine and pyrene along with 2-3 drops of MeOH was ground in a mortar with a pestle for 15 minutes. Diffraction quality block shaped red colour crystals were obtained from THF at room temperature after 3-4 days.

4,6-Dichlororesorcinol.2,5-Dimethylpyrazine.Pyrene (31): A 1:1:2 mixture of 4,6-dichlororesorcinol, 2,5-dimethylpyrazine and pyrene along with 2-3 drops of MeOH was ground in a mortar with a pestle for 15 minutes. Diffraction quality block shaped colourless crystals were obtained from MeCN at room temperature after 3-4 days.

4,6-Dichlororesorcinol.Tetramethylpyrazine.Phenazine.Pyrene (32): A 2:1:1:1 mixture of 4,6-dichlororesorcinol, tetramethylpyrazine, phenazine and pyrene along with 2-3 drops of MeOH was ground in a mortar with a pestle for 15 minutes. Diffraction quality block shaped yellow colour crystals were obtained from 1:1 EtOH:Me_2CO at room temperature after 3-4 days.

S2. Characterization techniques

Single crystal X-ray data were collected on a Rigaku Mercury 375/M CCD (XtaLAB mini) diffractometer using graphite monochromator Mo-Kα radiation and were processed with Rigaku crystal clear software (Rigaku, 2009). Data integration and data reduction were carried out using the SAINTPLUS program. The structures were solved by SHELXS-2017 (Sheldrick, 2008) using direct methods embedded in the WinGX suite (Farrugia, 2012) of programs. Full matrix least-squares refinements were carried out on F^2 for all non-hydrogen atoms using SHELXL-2017 (Sheldrick, 2015) with anisotropic displacement parameters. The hydrogen atoms were added for all the atoms either from difference Fourier maps or in their calculated positions using the riding model. Mercury version 4.1.3 (Macrae et al., 2008) was used for molecular representations. Crystallographic data are given in the Supporting Information, (Table S1). Structural data are available at CCDC 1989214-1989224, 2026215-2026234 and 2039064.

Farrugia, L. J. (2012). *J. Appl. Cryst.* 45, 849-854.

Macrae, C. F., Bruno, I. J., Chisholm, J. A., Edgington, P. R., McCabe, P., Pidcock, E., Rodriguez Monge, L., Taylor, R., van de Streek, J. & Wood, P. A. (2008). *J. Appl. Crystallogr.* 41, 466–470.

Rigaku (2009). *Crystal Clear-SM Expert 2.0.* Rigaku Corporation, Tokyo, Japan.

Sheldrick, G. M. (2008). *Acta Crystallogr. Sect. A.* 64, 112–122.

Sheldrick, G. M. (2015). *Acta Crystallogr. Sect. C.* 71, 3–8.

S3. Description of the two pseudopolymorphic binary cocrystals NRES:TMP

The binary cocrystal NRES:TMP was obtained in two pseudopolymorphic forms. The first is a 2:1 form that crystallizes in the non-centrosymmetric space group P1. The second is a 2:3 form which crystallizes in the centrosymmetric space group P-1. The connectivity (topology) of TMP and NRES molecules is the same in both forms (Figure S1). The packing diagram of two cocrystals is shown in Figure S2.

NRES: TMP(Form I):

The NRES:TMP (Form I) cocrystal is obtained in space group P1. An adequate search for higher symmetry (P-1) was carried out to confirm the non-centrosymmetric space group. These precautions were taken because the TMP molecules are located on local pseudo-centres of symmetry in the P1 structure (Desiraju *et al.*, 1991). Further checking of any purported P-1 structure for this form with the PLATON and the ADDSYM routines suggested no obvious space group change from P1 to P-1.

In this 2:1 form, the two NRES molecules have different conformations. In one, the two hydroxyl groups are in the *syn-syn* conformation and the nitro group is twisted nearly perpendicular to the aromatic ring, and the hydroxyl groups are intermolecularly O−H...N hydrogen bonded to the TMP molecules. In the other, the two hydroxyl groups are in the *anti-anti* conformation and are intramolecularly hydrogen bonded to the nitro group which is therefore coplanar with the aromatic ring.

Desiraju, G. R., Calabrese, J. C. & Harlow, R. L. (1991). *Acta Crystallogr. Sect. B*. **47**, 77–86.

NRES: TMP (Form II):

NRES: TMP (Form II) crystallizes in space group P-1. In this 2:3 form, one of the three TMP molecules is connected to NRES with O−H...N hydrogen bonds only to form an infinite running chain. The other two are additionally involved in weak C−H...O and C−H...π interactions with an adjacent NRES molecule.

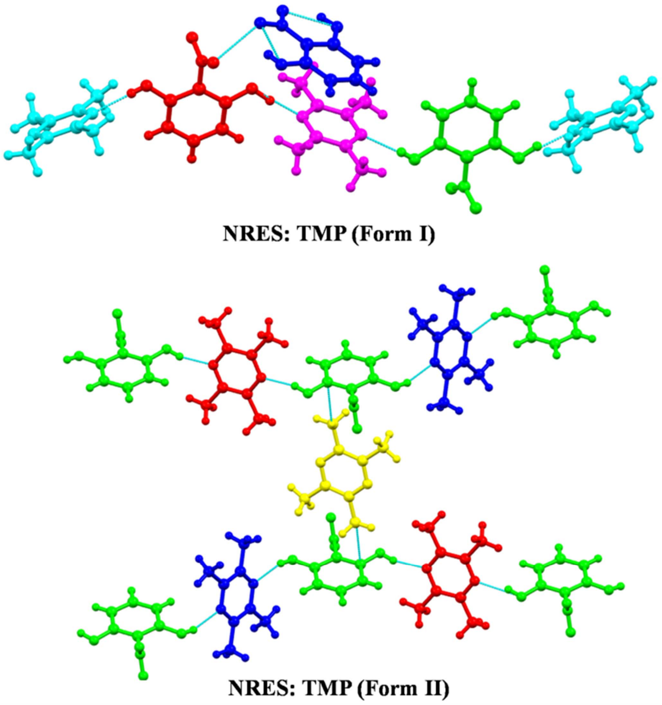

Figure S1 The connectivity of NRES and TMP molecules in NRES:TMP (form I) and NRES:TMP (form II). Note: TMP molecules are present in two different environment showing in different colours.

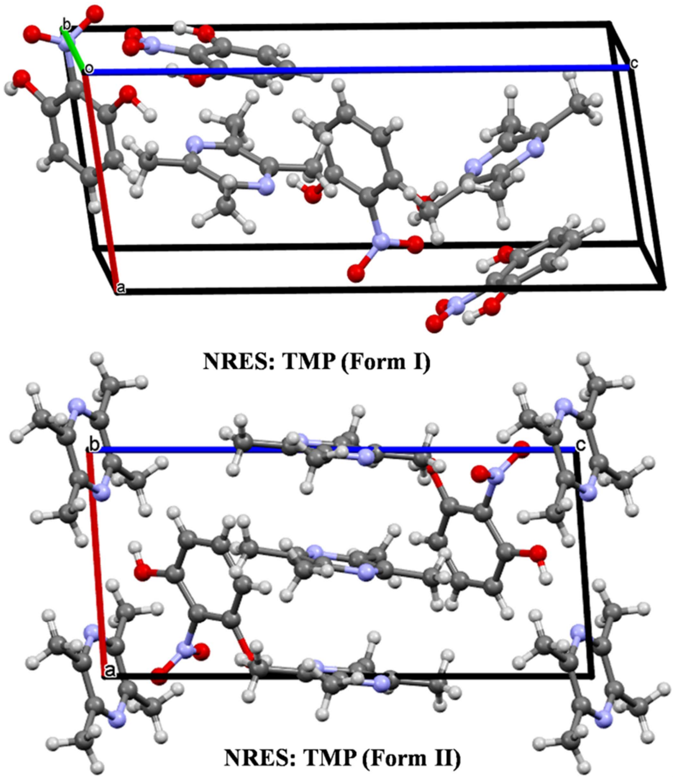

Figure S2 Packing diagram of NRES:TMP (form I) and NRES:TMP (form II).

Table S1 Crystallographic tables

**Compound**	1	2	3	4	5	6
**CCDC No.**	1989214	2039064	1989215	1989216	1989217	1989218
**Mol.Formula**	$C_{20}H_{22}N_4O_8$	$C_{18}H_{23}N_4O_4$	$C_{12}H_{13}N_3O_4$	$C_{32}H_{23}N_3O_4$	$C_{19}H_{15}N_3O_4$	$C_{26}H_{21}N_5O_4$
**Formula weight**	446.41	359.40	263.25	513.53	349.34	467.48
**T (K)**	150(2)	150(2)	150(2)	150(2)	150(2)	150(2)
**Crystal system**	Triclinic	Triclinic	Monoclinic	Triclinic	Triclinic	Triclinic
**Space group**	*P1*	*P-1*	C_2/c	*P-1*	*P-1*	*P-1*
**a (Å)**	7.7162(8)	7.658(2)	8.7482(14)	9.2447(9)	7.7638(14)	7.3217(8)
**b (Å)**	8.9727(9)	9.277(3)	14.926(3)	14.4694(13)	8.5897(15)	11.6513(13)
**c (Å)**	16.9089(17)	15.152(5)	10.2464(17)	19.3202(18)	12.723(2)	13.9987(16)
**α (°)**	86.559(6)	79.304	90	93.232(7)	105.585(7)	105.915(7)
**β (°)**	78.838(6)	82.008	110.165(8)	102.634(7)	102.978(7)	102.665(7)
**γ (°)**	66.314(5)	65.214	90	97.795(7)	95.062(7)	91.242(6)
**V(Å^3)**	1051.59(19)	957.9(5)	1255.9(4)	2488.6(4)	786.3(2)	1116.2(2)
**Z**	2	2	4	4	2	2
**ρcalc (g/cm^3)**	1.410	1.246	1.392	1.371	1.475	1.391
**μ (mm^{-1})**	0.111	0.090	0.107	0.092	0.106	0.097
**F(000)**	468	382	552	1072	364	488
**Total Reflns.**	10971	6882	6402	25721	7168	11717
**Unique Reflns.**	9240	3305	1441	11355	3538	5120
**Comple. (%)**	99.8	98.7	99.7	99.8	98.8	99.9
**R_{int}**	0.0171	0.0589	0.0646	0.0450	0.0391	0.0232
**R_1 (F^2)**	0.0477	0.0781	0.0566	0.0544	0.0530	0.0397
**wR_2(F^2)**	0.1004	0.2296	0.1620	0.1332	0.1414	0.0945

**Compound**	7	8	9	10	11
**CCDC No.**	1989219	1989220	1989221	1989222	1989223
**Mol.Formula**	$C_{42}H_{34}N_8O_8$	$C_{18}H_{17}N_3O_4$	$C_{18}H_{15}N_3O_4$	$C_{16}H_{13}N_5O_4$	$C_{20}H_{25}N_5O_4$
**Formula weight**	778.77	339.34	337.33	339.31	399.45
**T (K)**	150(2)	150(2)	150(2)	150(2)	150(2)
**Crystal system**	Triclinic	Monoclinic	Monoclinic	Monoclinic	Triclinic
**Space group**	*P-1*	$P2_1/c$	$P2_1/c$	$P2_1/c$	*P-1*
**a (Å)**	7.4323(10)	17.802(3)	17.9122(13)	18.213(3)	7.7563(12)
**b (Å)**	9.1138(12)	7.5355(11)	7.4259(5)	7.2967(13)	8.1082(13)
**c (Å)**	14.562(2)	12.5671(19)	12.1765(8)	11.940(2)	16.240(3)
**α (°)**	106.397(7)	90	90	90	97.387(7)
**β (°)**	103.133(7)	91.187(6)	92.337(7)	92.967(7)	95.164(7)
**γ (°)**	90.486(6)	90	90	90	95.007(7)
**V(Å^3)**	918.7(2)	1685.5(4)	1618.30(19)	1584.7(5)	1003.7(3)
**Z**	1	4	4	4	2
***ρ*calc (g/cm3)**	1.408	1.337	1.385	1.422	1.322
**μ(mm^{-1})**	0.100	0.096	0.100	0.106	0.094
**F(000)**	406	712	704	704	424
**Total Reflns.**	9526	16334	16284	14469	10336
**Unique Reflns.**	4200	3859	3707	3639	4570
**Comple. (%)**	99.8	99.9	99.9	99.8	99.8
**R_{int}**	0.0236	0.0737	0.0403	0.0544	0.0255
**R_1 (F^2)**	0.0453	0.0736	0.0425	0.0577	0.0557
**wR_2(F^2)**	0.1266	0.1832	0.1008	0.1393	0.1332

**Compound**	12	13	14	15	16
**CCDC No.**	1989224	2026215	2026216	2026217	2026218
**Mol. Formula**	$C_{13}H_{15}N_3O_4$	$C_{29}H_{26}N_3O_4$	$C_{58}H_{50}N_6O8$	$C_{40}H_{46}N_8O_8$	$C_{38}H_{42}N_8O_8$
**Formula weight**	277.28	962.97	1918.07	766.85	738.79
**T (K)**	150(2)	150(2)	150(2)	150(2)	150(2)
**Crystal system**	Monoclinic	Monoclinic	Triclinic	Triclinic	Triclinic
**Space group**	$P2_1/n$	$P2_1$	*P-1*	*P-1*	*P-1*
**a (Å)**	10.1301(13)	7.7925(13)	13.337(3)	7.5319(7)	7.4104(7)
**b(Å)**	12.6967(17)	12.885(2)	13.504(3)	8.6425(8)	8.8964(9)
**c(Å)**	10.3338(14)	12.398(2)	16.069(3)	15.3925(14)	15.7722(15)
**α (°)**	90	90	75.831(5)	78.729(6)	95.531(7)
**β (°)**	99.170(7)	104.054	66.422(5)	75.782(5)	101.446(7)
**γ (°)**	90	90	67.934(5)	89.099(6)	111.676(8)
**V(Å^3)**	1312.1(3)	1207.6(4)	2442.9(8)	951.99(15)	930.47(16)
**Z**	4	2	2	1	1
**ρcalc (g/cm^3)**	1.404	1.322	1.304	1.338	1.318
**μ(mm^{-1})**	0.106	0.089	0.088	0.095	0.095
**F(000)**	584	506	1008	406	390
**Total Reflns.**	13192	12163	25588	9956	9757
**Unique Reflns.**	3009	5506	11144	4348	4270
**Comple. (%)**	99.9	99.8	99.8	99.8	99.7
**R_{int}**	0.0273	0.0572	0.0621	0.0225	0.0270
**$R_1(F^2)$**	0.0382	0.0905	0.0873	0.0432	0.0517
**$wR_2(F^2)$**	0.0898	0.2407	0.2244	0.1140	0.1361

**Compound**	17	18	19	20	21
**CCDC No.**	2026219	2026220	2026221	2026222	2026223
**Mol. Formula**	$C_{22}H_{23}N_3O_4S_2$	$C_{62}H_{50}N_6O_8$	$C_{50}H_{52}N_8O_8$	$C_{42}H_{42}N_8O_8$	$C_{42}H_{40}N_8O_8$
**Formula weight**	915.11	1007.08	892.99	786.83	784.82
**T (K)**	150(2)	150(2)	150(2)	150(2)	150(2)
**Crystal system**	Triclinic	Triclinic	Triclinic	Triclinic	Triclinic
**Space group**	*P-1*	*P-1*	*P-1*	*P-1*	*P-1*
**a (Å)**	7.6512(11)	7.6840(18)	7.7634(12)	7.5543(17)	7.504(3)
**b(Å)**	8.9495(13)	9.266(2)	8.9486(14)	8.914(2)	9.428(4)
**c(Å)**	18.121(3)	18.043(4)	16.368(3)	14.713(3)	14.102(6) Å
**α (°)**	87.883(6)	96.343(7)	83.021(6)	85.797(7)	94.052(8)
**β (°)**	78.725(6)	94.419(7)	82.842(6)	88.497(6)	91.192(7)
**γ (°)**	65.728(5)	107.054(7)	88.111(6)	89.487(6)	90.498(8)
**V($Å^3$)**	1108.0(3)	1212.6(5)	1119.7(3)	987.7(4)	995.0(7)
**Z**	2	1	1	1	1
**ρcalc (g/cm^3)**	1.371	1.379	1.324	1.323	1.310
**μ(mm^{-1})**	0.274	0.093	0.091	0.094	0.093
**F(000)**	480	528	472	414	412
**Total Reflns.**	11118	12080	11576	10148	7618
**Unique Reflns.**	5059	5501	5114	4515	3479
**Comple. (%)**	99.7	99.8	99.8	99.8	99.3
**R_{int}**	0.0385	0.0924	0.0397	0.0425	0.1474
**R_1 (F^2)**	0.0671	0.0855	0.0630	0.0629	0.0931
**wR_2(F^2)**	0.1891	0.2084	0.1944	0.1624	0.1896

Compound	22	23	24	25	26	27
CCDC No.	2026224	2026225	2026226	2026227	2026228	2026229
Mol. Formula	$C_{40}H_{40}NOS_2$	$C_{40}H_{38}NS_2$	$C_{41}H_{41}N_7O_8S$	$C_{41}H_{39}N_7O_8S$	$C_{14}H_{16}Cl_2N_2O_2$	$C_{36}H_{24}Cl_2N_5O_2$
Formula weight	796.90	794.88	791.87	789.85	315.19	629.50
T (K)	150(2)	150(2)	150(2)	150(2)	150(2)	150(2)
Crystal system	Triclinic	Triclinic	Triclinic	Triclinic	Monoclinic	Triclinic
Space group	*P-1*	*P-1*	*P-1*	*P-1*	$P2_1/n$	*P-1*
a (Å)	7.489(3)	7.514(3)	7.4741(15)	7.4078(12)	9.302(3)	10.807(5)
b (Å)	9.107(3)	9.272(3)	9.2215(19)	9.3192(15)	9.394(2)	12.145(5)
c (Å)	14.187(5)	13.852(5)	14.295(3)	13.815(2)	17.660(6)	12.466(6)
α (°)	97.784(7)	83.832(7)	96.245(7)	94.239(6)	90	108.220(8)
β (°)	93.611(7)	89.887(7)	92.609(7)	91.445(6)	104.075(16)	104.061(7)
γ (°)	90.872(6)	88.173(7)	92.314(7)	90.299(6)	90	101.072(7)
V ($Å^3$)	956.5(6)	958.9(6)	977.4(3)	950.8(3)	1497.0(4)	1442.3(11)
Z	1	1	1	1	4	2
ρcalc (g/cm^3)	1.383	1.376	1.345	1.379	1.398	1.449
μ (mm^{-1})	0.201	0.201	0.146	0.150	0.436	0.270
F(000)	418	416	416	414	656	650
Total Reflns.	8562	7607	9903	9734	15055	11846
Unique Reflns.	4278	3352	4424	4336	3400	6333
Comple. (%)	98.4	99.7	99.8	99.7	100.0	99.9
R_{int}	0.0861	0.0603	0.0506	0.0442	0.1507	0.1036
R_1 (F^2)	0.0890	0.0784	0.0697	0.0580	0.0791	0.0880
wR_2 (F^2)	0.2229	0.2175	0.1577	0.1265	0.1969	0.2076

**Compound**	28	29	30	31	32
**CCDC No.**	2026230	2026231	2026232	2026233	2026234
**Mol. Formula**	$C_{12}H_{12}Cl_2N_2O_2$	$C_{44}H_{42}Cl_4N_4O_4$	$C_{84}H_{54}Cl_4N_4O_4$	$C_{28}H_{22}Cl_2N_2O_2$	$C_{80}H_{66}Cl_8N_8O_8$
**Formula weight**	287.14	832.61	1325.11	489.37	1551.00
**T (K)**	150(2)	150(2)	150(2)	150(2)	150(2)
**Crystal system**	Monoclinic	Monoclinic	Monoclinic	Triclinic	Monoclinic
**Space group**	$P2_1/c$	C_2/c	C_2/c	*P-1*	$P2_1$
**a (Å)**	8.188(12)	42.727(11)	33.873(7)	8.8453(13)	17.219(5)
**b(Å)**	15.73(2)	8.899(3)	10.374(2)	10.4891(15)	22.693(7)
**c(Å)**	11.247(16)	22.920(6)	18.332(4)	12.6940(19)	18.331(5)
**α (°)**	90	90	90	94.129(7)	90
**β (°)**	110.491(16)	114.062(8)	91.186(6)	94.866(7)	91.312(6)
**γ (°)**	90	90	90	98.239(7)	90
**V($Å^3$)**	1357(3)	7958(4)	6441(6)	1157.1(3)	7161(4)
**Z**	4	8	4	2	4
**ρcalc (g/cm^3)**	1.406	1.390	1.367	1.405	1.439
**μ(mm^{-1})**	0.473	0.347	0.243	0.310	0.380
**F(000)**	592	3472	2744	508	3208
**Total Reflns.**	7471	37117	32417	12258	59375
**Unique Reflns.**	3075	9097	7363	5291	30424
**Comple. (%)**	99.0	99.8	99.8	99.8	99.6
**R_{int}**	0.1147	0.0818	0.1362	0.0323	0.1068
**$R_1(F^2)$**	0.0666	0.0525	0.0776	0.0428	0.1229
**$wR_2(F^2)$**	0.1824	0.1038	0.1610	0.1188	0.3145

S4. GC-mass spectroscopy

The GC-mass spectra of the multi-component molecular solids (**24** and **25**) were recorded from methanolic solutions of the corresponding single crystals. The cell parameters of the single crystals were confirmed before the experiment. The data clearly revealed the existence of five different organic molecules in **24** and **25**. Therefore, the crystals of **24** and **25** are quinary molecular solids. The GC-mass spectrum of **24** indicates five distinct ion peaks at m/z = 136 (for tetramethylpyrazine), 109 (for 2- nitroresorcinol), 166 (for 2,2'-bithiophene), 156 (for 2,2'-bipyridine) and at 184 (for 1,2-bis(4-pyridyl)ethane) whereas in **25** we observed ion peaks at m/z = 136 (for tetramethylpyrazine), 109 (for 2- nitroresorcinol), 166 (for 2,2'-bithiophene), 156 (for 2,2'-bipyridine) and at 181 (for 1,2-bis(4-pyridyl)ethene).

Figure S3 GC-mass spectrum of 24

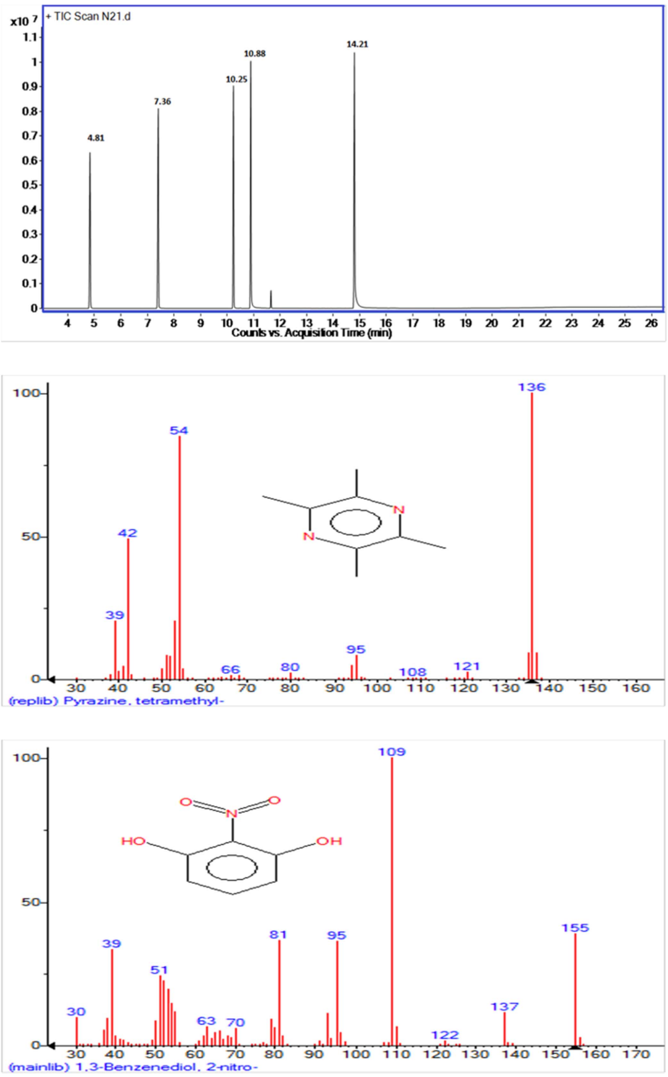

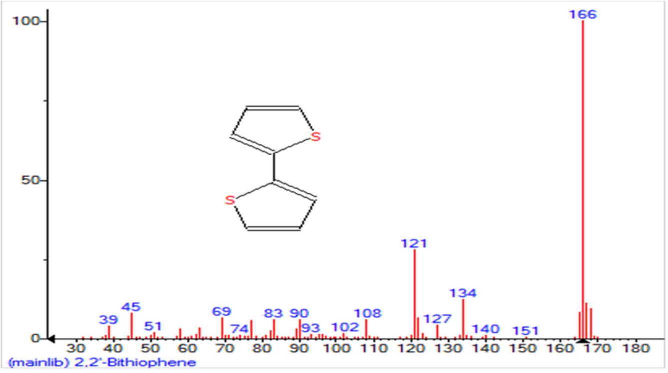

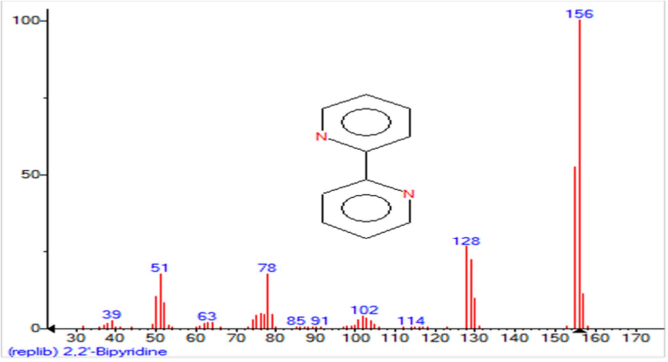

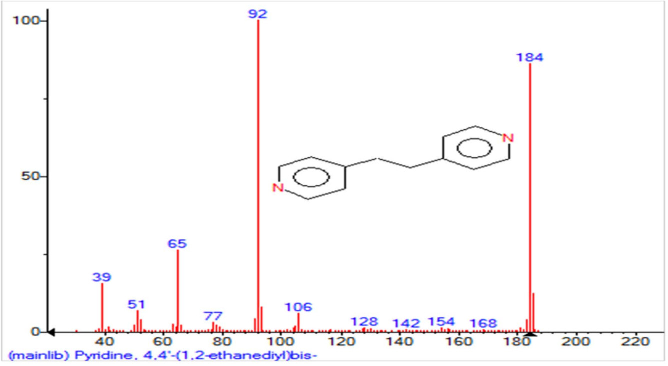

Figure S4 GC-mass spectrum of **25**

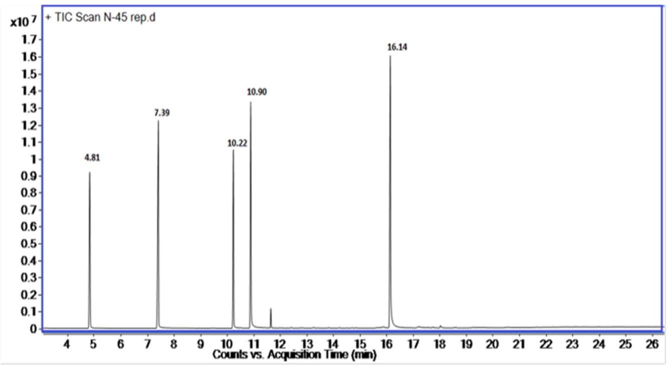

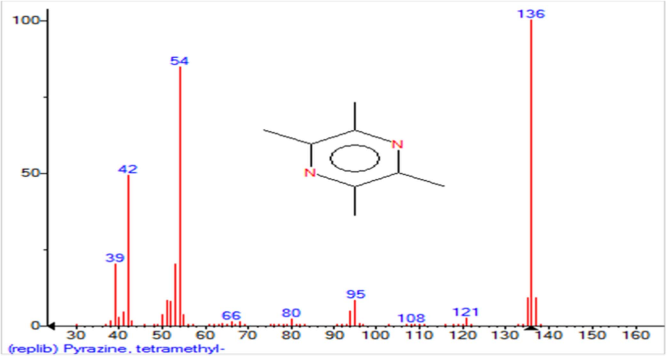
136
54
42
39
66
80
95
108
121
N
N
(replib) Pyrazine, tetramethyl-

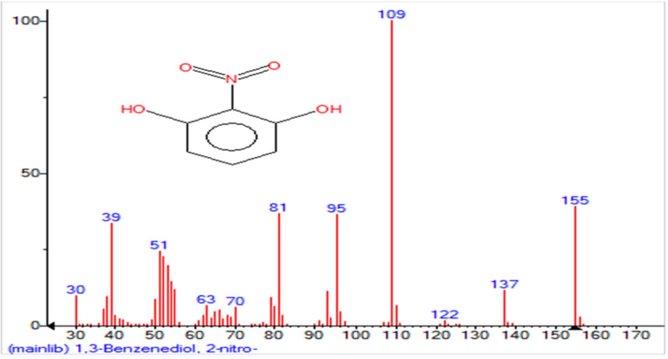
109
O
N
O
HO
OH
81
95
155
39
51
30
63
70
137
122
(mainlib) 1,3-Benzenediol, 2-nitro-

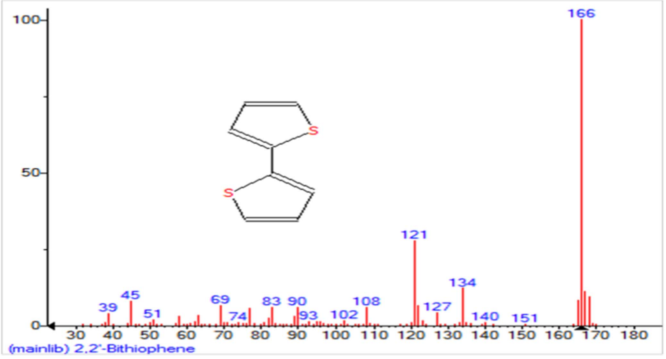
166
S
S
121
134
39
45
51
69
74
83
90
93
102
108
127
140
151
(mainlib) 2,2'-Bithiophene

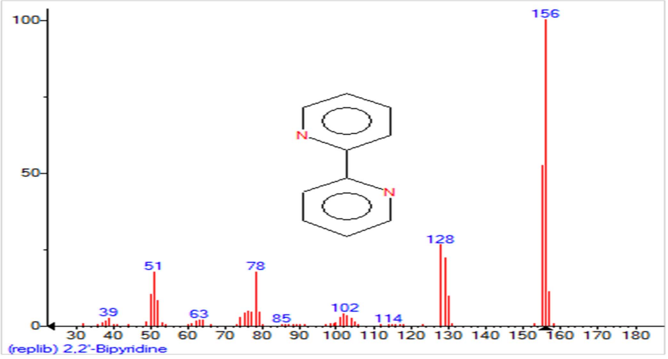
156
N
N
128
51
78
39
63
85
102
114
(replib) 2,2'-Bipyridine

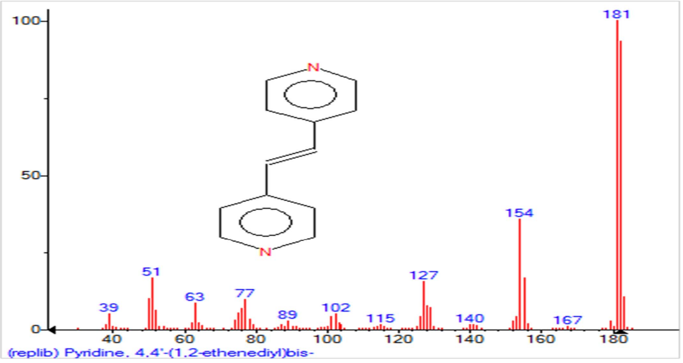

Table S2 Unsuccessful (co)crystallization experiments

Compound	Radio	Solvent	Results
NRES:TMP:ACR:PERY	1:1:1:1	MeOH, THF and MeCN	NRES:TMP
NRES:TMP:ACR:HMB	1:1:1:1	MeOH, THF and MeCN	NRES:TMP
NRES:TMP:ACR:ANT	1:1:1:1	MeOH, THF and MeCN	NRES:TMP
NRES:TMP:ACR:22BP	1:1:1:1	MeCN	NRES:TMP:22BP
NRES:TMP:ACR:22TBP	1:1:1:1	MeCN	NRES:TMP:22TBP
NRES:TMP:PYR:DPE-I	1:1:1:1	$MeNO_2$ and MeCN	NRES:TMP:DPE-I
NRES:DMP:PYR:DPE-I	1:1:1:1	THF	NRES:DPE-I
NRES:TMP:PYR:DPE-II	1:1:1:1	MeCN	NRES:DPE-II
NRES:TMP:PYR:PHE	1:1:1:1	MeOH, THF and MeCN	NRES:TMP
NRES:DMP:PYR:4DMAP	1:1:1:1	MeCN	NRES:PYR:4DMAP
NRES:TMP:PYR:44BP	1:1:1:1	MeOH, THF and MeCN	NRES:44BP
NRES:TMP:PYR:4PP	1:1:1:1	$MeNO_2$	NRES:TMP:PYR
NRES:TMP:22BP:DPP-I	2:1:1:1	MeCN	NRES:22BP:DPP-I
NRES:TMP:33BP:DPE-I	2:1:1:1	MeOH	NRES:33BP
